# Protein *Trans*-Splicing of Multiple Atypical Split Inteins Engineered from Natural Inteins

**DOI:** 10.1371/journal.pone.0059516

**Published:** 2013-04-08

**Authors:** Ying Lin, Mengmeng Li, Huiling Song, Lingling Xu, Qing Meng, Xiang-Qin Liu

**Affiliations:** 1 Institute of Biological Sciences and Biotechnology, Donghua University, Shanghai, P.R. China; 2 Department of Biochemistry and Molecular Biology, Dalhousie University, Halifax, Nova Scotia, Canada; Center for Genomic Regulation, Spain

## Abstract

Protein *trans*-splicing by split inteins has many uses in protein production and research. Splicing proteins with synthetic peptides, which employs atypical split inteins, is particularly useful for site-specific protein modifications and labeling, because the synthetic peptide can be made to contain a variety of unnatural amino acids and chemical modifications. For this purpose, atypical split inteins need to be engineered to have a small N-intein or C-intein fragment that can be more easily included in a synthetic peptide that also contains a small extein to be *trans*-spliced onto target proteins. Here we have successfully engineered multiple atypical split inteins capable of protein *trans*-splicing, by modifying and testing more than a dozen natural inteins. These included both S1 split inteins having a very small (11–12 aa) N-intein fragment and S11 split inteins having a very small (6 aa) C-intein fragment. Four of the new S1 and S11 split inteins showed high efficiencies (85–100%) of protein *trans*-splicing both in *E. coli* cells and *in vitro*. Under *in vitro* conditions, they exhibited reaction rate constants ranging from ∼1.7×10^−4^ s^−1^ to ∼3.8×10^−4^ s^−1^, which are comparable to or higher than those of previously reported atypical split inteins. These findings should facilitate a more general use of *trans*-splicing between proteins and synthetic peptides, by expanding the availability of different atypical split inteins. They also have implications on understanding the structure-function relationship of atypical split inteins, particularly in terms of intein fragment complementation.

## Introduction

Inteins are internal protein elements that self-excise from their host protein and catalyze ligation of the flanking sequences (exteins) with a peptide bond [1]. Over 600 inteins have been found in organisms of all three domains of life and in various host proteins including viral proteins [2]. A bi-functional intein has a homing endonuclease domain inside a splicing domain, but the endonuclease domain may be deleted without impairing the protein splicing function [3]. A mini-intein has a contiguous splicing domain only, whereas a split intein consists of two intein fragments [4]. Different mini-inteins (or the splicing domains of bi-functional inteins) showed very similar crystal structures [5]–[8], although they may have low levels of sequence identity. The crystal structure of a mini-intein consists of ∼12 β-strands that form a disc-shaped protein, with the splice junctions in the centrally located catalytic pocket.

Split inteins are capable of protein *trans*-splicing, where one precursor protein consists of a polypeptide (N-extein) fused to the N-terminal intein fragment (N-intein), and another precursor protein consists of the C-terminal intein fragment (C-intein) fused to another polypeptide (C-extein). Upon *trans*-splicing of the two precursor proteins, the intein fragments self-excise, and the N-extein and the C-extein are joined with a peptide bond. Protein splicing or *trans*-splicing has found many uses including production of cytotoxic proteins [9], segmental isotope labeling of proteins for NMR studies [10], gene therapy procedure using split genes [11], transgenic plants to prevent environmental escape of the transgene [12], [13], protein two-hybrid methods for detecting protein-protein interactions and sub-cellular protein localization [14], [15], site-specific protein labeling and modifications [16]. A small number of split inteins have been found in nature, and additional split inteins have been engineered from bi-functional inteins and mini-inteins by deleting the endonuclease domain (if present) and splitting the splicing domain into two fragments [17]–[20]. In these conventional split inteins, whether natural or engineered, the split site is relatively close to the middle of the intein sequence and corresponds to location of the homing endonuclease domain. They have an N-terminal fragment of ∼100 aa long and a C-terminal fragment of ∼40 aa long and were referred to as S0 split inteins [21]. These intein fragments’ relatively large sizes make it difficult to produce them in synthetic peptides that also need to contain desired extein sequences.

An atypical S1 split intein has been engineered from an *Ssp* DnaB mini-intein by splitting the intein sequence at a site proximal to the N-terminal, producing an N-terminal fragment (N-intein) of only 11 aa in length and a C-terminal fragment (C-intein) of 144 aa in length [21]. The small N-intein allowed *trans*-splicing of synthetic peptides onto the N-terminus of recombinant proteins, with the synthetic peptide carrying chemical labeling or modifications [22]. More recently, an atypical S11 split intein was engineered from a *Ssp* GyrB mini-intein by splitting the intein sequence at a site proximal to the C-terminal, producing a C-terminal fragment (C-intein) of only 6 aa in length and an N-terminal fragment (N-intein) of 150 aa in length [23]. The small C-intein facilitated *trans*-splicing of synthetic peptides onto the C-terminus of recombinant proteins, with the synthetic peptide carrying desired chemical labeling or modifications [24].

For more general uses of *trans*-splicing between proteins and synthetic peptides, it is highly desirable to produce additional atypical split inteins (S1 and S11 types) that can *trans*-splice *in vivo* and *in vitro.* This is because different inteins often work at different efficiencies when used in different host proteins having different amino acid residues flanking the splice sites, therefore an increased availability of different atypical split inteins may allow people to choose an intein that works most efficiently with a particular host protein of interest. However, it was not clear whether additional atypical split inteins could be produced, because some previous attempts of engineering additional S1 and S11 split inteins had failed [21]. An S11 split intein derived from an *Ssp* DnaB mini-intein failed to *trans*-splice, and a large C-intein of an S1 split intein derived from *Ssp* DnaX intein underwent spontaneous C-cleavage [25]. In this study, we systematically modified and tested a large number of natural inteins, in order to produce new atypical split inteins capable of protein *trans*-splicing. We successfully produced both S1 split inteins and S11 split inteins, with each intein showing efficient *trans*-splicing in *E. coli* cells and *in vitro*. Reaction rate constants were also determined through kinetic analysis of *in vitro trans*-splicing, and they were found to be either comparable to or significantly higher than those of previously described S1 and S11 split inteins. These findings significantly increased availability of atypical split inteins that can be particularly useful for site-specific protein labeling or modifications through protein-peptide *trans*-splicing. They also provided insights on intein structure-function and fragment complementation, which can be useful for future efforts to engineer atypical split inteins.

## Materials and Methods

### Construction of Mini- and Split Inteins

Intein sequences (both protein and DNA) were retrieved from the intein database at http://www.neb.com/neb/inteins.html [2]. Protein sequence alignments were carried out using the ClustalW program online [26]. Intein coding sequences were prepared by PCR and inserted in the pMST plasmid between *Xho* I and *Age* I sites [21]. To construct mini-intein plasmids, inverse PCR was used as previously described [25] to delete coding sequences of putative endonuclease domain (if present), which also inserted coding sequence of a linker peptide (ASGHHHHHHGGSGS) at the site of deletion.

To construct split intein plasmids for protein *trans*-splicing in *E. coli*, a spacer sequence was inserted in the mini-intein coding sequence at the split site by inverse PCR as described previously [25]. This spacer DNA sequentially contains a stop codon, a ribosome binding site, and a start codon. This creates a two-gene operon, where the first gene encodes the N-protein consisting of a maltose-binding protein and N-intein, while the second gene encodes the C-protein consisting of the C-intein and thioredoxin.

For *in vitro trans*-splicing, plasmids were constructed to express either the N-protein or the C-protein individually. To construct plasmids expressing only the N-protein, the C-protein coding sequence in the split intein plasmid was deleted between *Afl* II and *Hind* III sites, leaving only the N-protein coding sequence. To construct plasmids expressing only the C-protein for S1 split inteins, the C-protein coding sequence was isolated from the split intein plasmid as an *Nde* I-*Pst* I fragment and inserted into pTWIN1 plasmid (New England Biolabs) between the same two sites. For S11 split inteins, the C-protein coding sequence was prepared by PCR from the split intein plasmid, digested with *Nde* I and *Nhe* I, and inserted in pET-32a plasmid (Novagen) between the same two sites, which also added a hexahistidine tag to the C-terminus of the C-protein. All relevant DNA sequences were verified through DNA sequencing.

### Protein Expression and Splicing in E. coli Cells

zEach recombinant plasmid was introduced into *E. coli* DH5α strain using standard transformation methods. The resulting *E. coli* cells were grown in liquid LB medium containing ampicillin (50 µg/ml) to log phase (A_600_≈0.6), and IPTG was added to a final concentration of 0.8 mM to induce protein expression at room temperature for overnight. Cells were harvested by centrifugation and lysed in a standard SDS-containing gel-loading buffer in a boiling water bath for 10 min. Electrophoresis was performed in 12% SDS–polyacrylamide gels. Western blotting was performed using anti-thioredoxin antibody and the WesternBreeze™ Immunodetection Kit (Invitrogen) as recommended by the manufacturer.

### In vitro Protein Trans-splicing

The N-protein was expressed in *E. coli* as above and affinity purified using amylose resin according to manufacturer’s instructions (New England Biolabs). The C-protein was expressed in *E. coli* BL21(DE3) strain as above and affinity purified using Ni-NTA resin according to manufacturer’s instructions (Qiagen). Purified N-protein and C-protein were mixed in a specified molar ratio and incubated at indicated temperature for specified time. Reaction was stopped by adding SDS-PAGE loading buffer, and analyzed by SDS-PAGE followed by Commassie blue staining or Western blotting using anti-thioredoxin antibody (Invitrogen).

## Results

### Construction and Test of Mini-inteins

**Figure 1:**
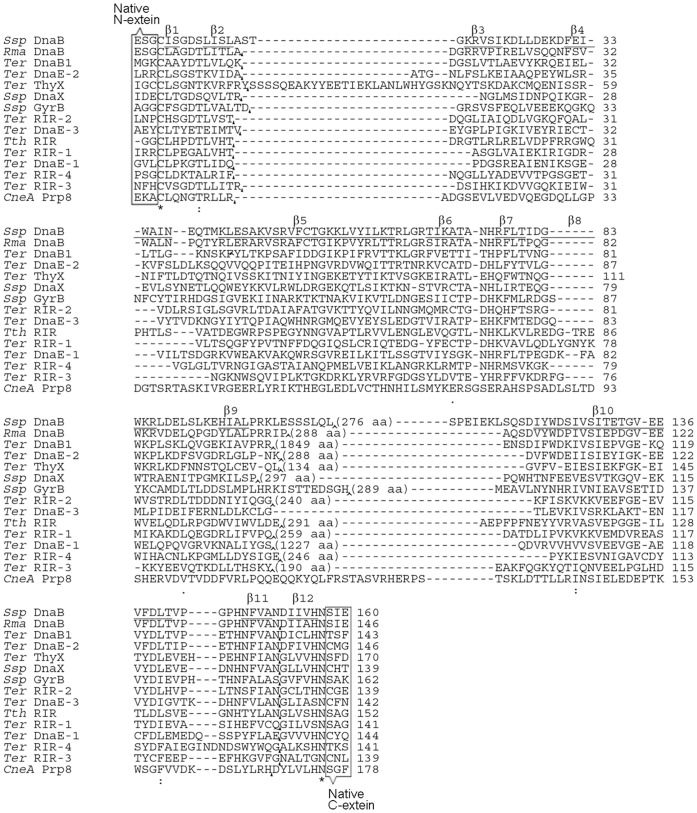
Amino acid sequences of mini-inteins and split inteins. Mini-intein sequences are aligned using ClustalW online [26], and gaps (represented by -) were introduced to optimize the alignment. *CneA* PRP8 intein was a natural mini-intein. *Ter* DnaE-3 mini-intein was derived from a natural conventional split intein by a fusion of the intein fragments. Other mini-inteins were derived from natural inteins by a deletion of their putative endonuclease domain sequences, with the position and number of deleted residues shown in parenthesis. A linker sequence (ASGHHHHHHGGSGS) was inserted at the site of deletion (or corresponding site in the *CneA* PRP8 and the *Ter* DnaE-3 mini-inteins) and marked with an arrowhead. For each intein, three (or two) amino acid residues (enclosed with a rectangle) of the native extein sequences on each side of the intein were included in all splicing studies. In the *Ssp* DnaB mini-intein, whose crystal structure is known, sequences of the 12 β-strands (β1 to β12) are underlined. Split sites for producing the S1 and S11 split inteins are marked with black triangles.

Although aiming for functional split inteins, we first wanted to convert natural inteins into functional mini-inteins by deleting putative endonuclease domain (if present), considering that the endonuclease domain is not needed for splicing and can even be detrimental for some applications. Fourteen natural inteins were selected because they were readily available to us, and because their splicing activities have been demonstrated previously in model proteins in *E. coli* [2]. These natural inteins were the *Rma* DnaB, *Ssp* DnaX, *Ter* ThyX, *Ter* DnaE-3, *Ter* DnaB1, *Ter* DnaE-1, *Ter* DnaE-2, *Ssp* GyrB, *Tth* RIR, *CneA* Prp8, *Ter* RIR-1, *Ter* RIR-2, *Ter* RIR-3 and *Ter* RIR-4 inteins. These intein names follow the standard intein nomenclature [1], [2], for example, with *Rma* DnaB intein being a natural intein found in the DnaB protein of *Rhodothermus marinus*. The *CneA* Prp8 intein was a natural mini-intein, the *Ter* DnaE-3 intein was naturally a conventional split intein, and the remaining 12 natural inteins all had contiguous sequences and putative (complete or partial) endonuclease domain sequences. Working from coding sequences of these inteins, putative endonuclease domain coding sequences (if present) were deleted to produce mini-inteins, using standard recombinant DNA techniques including inverse PCR as described previously [17]. The exact boundary for an endonuclease domain (if present) has been defined previously in the InBase intein database [2] and was based on previously described methods of intein domain predictions [2], [27], [28]. The resulting mini-intein sequences are shown in Figure 1, where all sequences are aligned with the *Ssp* DnaB and *Ssp* GyrB mini-intein sequences from which the previous S1 and S11 split inteins were derived [21], [23]. Considering that splicing activity may highly rely on the junction sequence, we flanked each intein sequence with 2–3 amino acid residues of its native extein sequences on each side of the intein, as shown in Figure 1. At the site of endonuclease domain deletion, a 14-aa linker peptide (ASGHHHHHHGGSGS) was inserted to provide structural flexibility and a hexahistidine tag that can be useful for protein identification and affinity purification.

**Figure 2:**
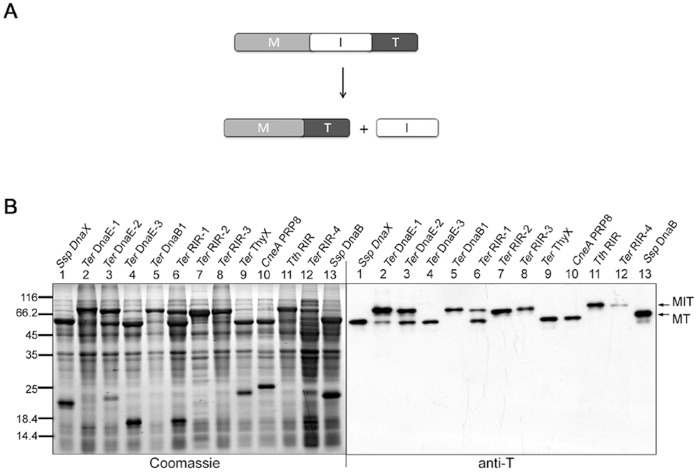
Mini-intein *cis*-splicing in *E. coli* cells. *A*. Schematic illustration of the *cis*-splicing reaction. The recombinant precursor protein consists of a maltose binding protein sequence (M) followed by the mini-intein sequence (I) and a thioredoxin sequence (T). *B*. Detection of splicing activity. After expression of the precursor protein containing a mini-intein (specified on top) in *E. coli* for overnight at 25°C, total cellular proteins were resolved by SDS-PAGE, followed by Western blotting using an anti-thioredoxin (anti-T) antibody that detected the precursor protein (MIT) and the spliced protein (MT).

The mini-intein constructs were expressed in *E. coli* using the previously described pMST plasmid [29]. As illustrated in Figure 2A, each intein (I) was flanked by a maltose binding protein (M) as the N-extein and a thioredoxin (T) as the C-extein, expressed from an IPTG-inducible P*tac* promoter. After the expression, total cellular proteins were resolved by SDS-PAGE and analyzed by Western blotting using an anti-T antibody to see the precursor protein (MIT) and possible spliced protein (MT). As shown in Figure 2B, nine of the tested mini-inteins produced the spliced protein, and their splicing efficiencies (percentage of the precursor protein that had spliced) ranged from ∼10% for the *Ter* DnaE-1 mini-intein to ∼100% for the *Ter* ThyX mini-intein. Splicing activities of the *CneA* PRP8, *Ssp* DnaX and *Ssp* DnaB mini-inteins were consistent with findings of other studies [23], [29], [30].

### Construction and Test of S1 Split Inteins for Trans-splicing in E. coli

**Figure 3:**
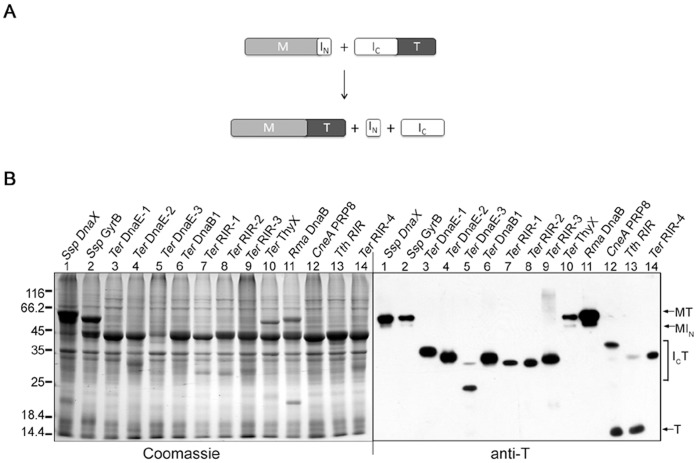
S1 split intein *trans*-splicing in *E. coli* cells. *A*. Schematic illustration of the *trans*-splicing reaction. Recombinant N-protein consists of a maltose binding protein sequence (M) followed by the small N-intein (I_N_). Recombinant C-protein consists of the larger C-intein (I_C_) followed by a thioredoxin (T). *B*. Detection of *trans*-splicing. For each S1 split intein (specified on top), the N-protein and the C-protein were co-expressed in *E. coli* for overnight at 25°C, total cellular proteins were resolved by SDS-PAGE, and protein bands were visualized by Coomassie Blue staining or by Western blotting using an anti-T antibody as indicated. Positions are marked for the spliced protein (MT), the N-protein (MI_N_), the C-protein (I_C_T), and a C-cleavage product (T).

Fourteen mini-inteins were converted into S1 type of split inteins by splitting the intein sequence at a site near the N-terminus, working through the coding DNA. As shown in Figure 1, the split site is located between two β-strands (β2 and β3), according to the *Ssp* DnaB mini-intein whose crystal structure has been determined [7]. In the resulting S1 split intein, the N-intein is 10 to 12 amino acids long, while the C-intein ranged from 122 to 162 aa in length. As illustrated in Figure 3A, the N-intein (I_N_) was fused to the C-terminus of a maltose binding protein (M) to create an N-protein, the C-intein (I_C_) was fused to the N-terminus of a thioredoxin to create a C-protein. The N-protein and the C-protein were co-expressed in *E. coli* from a 2-gene operon on a plasmid, which is similar to earlier studies of other split inteins [17], [18]. Total cellular proteins were resolved by SDS-PAGE and visualized by Coomassie blue staining and by Western blotting using an anti-T antibody (Figure 3B). Four of the fourteen S1 split inteins showed *trans*-splicing activity, as indicated by the accumulation of the spliced protein MT, and they are the *Rma* DnaB, *Ssp* DnaX, *Ssp* GyrB, and *Ter* ThyX S1 split inteins. On the Western blot that visualizes the C-protein (I_C_T) and the spliced protein (MT), these four split inteins showed little or no C-protein (I_C_T) accumulation, indicating that the *trans*-splicing reaction reached near completion and exhausted the C-protein. In the Coomassie blue stained picture that visualizes all proteins, a large amount of the N-protein remained not spliced, indicating that the N-protein was expressed in large excess over the C-protein that had been exhausted. This unequal expression was due to the fact that the N-protein gene was in front of the C-protein gene in the 2-gene operon, as had been observed previously [17], [18]. For the remaining ten S1 split inteins, no significant amount of the spliced protein (MT) was detected even on the Western blot, indicating an absence of *trans*-splicing. The *CneA* Prp8 and *Tth* RIR S1 split inteins showed more than 50% C-cleavage without *trans*-splicing, as indicated by the cleavage product T seen on the Western blot. *Trans*-splicing activity of the *Ssp* DnaX S1 split intein was also observed in another study [31].

### Construction and Test of S11 Split Inteins for Trans-splicing in E. coli

**Figure 4:**
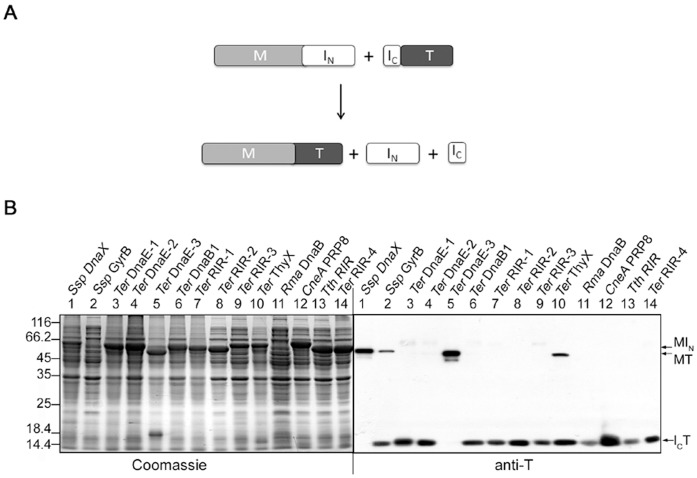
S11 split intein *trans*-splicing in *E. coli* cells. *A*. Schematic illustration of the *trans*-splicing reaction. Recombinant N-protein consists of a maltose binding protein sequence (M) followed by the large N-intein (I_N_). Recombinant C-protein consists of the small C-intein (I_C_) followed by a thioredoxin (T). *B*. Detection of *trans*-splicing. For each S11 split intein (specified on top), the N-protein and the C-protein were co-expressed in *E. coli* for overnight at 25°C, total cellular proteins were resolved by SDS-PAGE, and protein bands were visualized by Coomassie Blue staining or by Western blotting using an anti-T antibody as indicated. Positions are marked for the spliced protein (MT), the N-protein (MI_N_), and the C-protein (I_C_T).

Fourteen mini-inteins were converted into S11 type of split inteins by splitting the intein sequence at a site near the C-terminus, working through the intein coding DNA. The intein sequence was split at a site between β-strands β11 and β12 (see Figure 1), based on the *Ssp* DnaB mini-intein whose crystal structure has been determined [7]. In the resulting S11 split intein, the N-intein ranged from 127 to 165 aa in length, while the C-intein was just 6 to 7 amino acids long. As illustrated in Figure 4A, the N-intein (I_N_) was fused to a maltose binding protein (M) to create an N-protein, the C-intein (I_C_) was fused to thioredoxin to create a C-protein, and the two proteins were co-expressed in *E. coli* from a 2-gene operon as described above for the S1 split inteins. As seen in Figure 4B, total cellular proteins were resolved by SDS-PAGE, followed by Coomassie blue staining and by Western blotting using an anti-T antibody. Four of the fourteen S11 split inteins showed *trans*-splicing activity, as indicated by the accumulation of the spliced protein MT, and they are the *Ssp* DnaX, *Ssp* GyrB, *Ter* DnaE-3, and *Ter* ThyX S11 split inteins. Splicing efficiency (percentage of C-protein I_C_T that had been converted into the spliced protein MT) were estimated from the Western blot to be ∼100% for the *Ssp* DnaX S11 split intein, ∼40% for the *Ssp* GyrB S11 split intein, over 90% for the *Ter* DnaE-3 S11 split intein, and ∼35% for the *Ter* ThyX S11 split intein. *Trans*-splicing activity of the *Ssp* GyrB S11 split intein was lower than the >80% splicing efficiency found in a previous study [23], probably because the construct used in this study differs from the previous construct by having an embedded hexahistidine tag sequence. In the Coomassie blue stained picture, the spliced protein MT (if any) could not be readily identified, because it was not sufficiently separate from the over-expressed N-protein MI_N_. The remaining ten S11 split inteins did not show a significant amount of the spliced protein (MT) on the Western blot, indicating an absence of *trans*-splicing. For the *CneA* Prp8 S11 split intein, two protein bands were seen in the area of the C-protein on Western blot, with the larger one most likely being the C-protein I_C_T and the smaller one most likely being the C-cleavage product T.

### Protein Trans-splicing in vitro by S1 and S11 Split Inteins

Based on splicing activities observed in *E. coli*, two S1 split inteins (*Ssp* DnaX and *Ter* ThyX) and two S11 split inteins (*Ssp* DnaX and *Ter* DnaE-3) were chosen for further characterization *in vitro*. For each split intein, the N-protein and the C-protein were expressed separately in *E. coli*. The N-protein contained a maltose binding protein and was affinity-purified on amylose resin. The C-protein contained a hexahistidine tag and was affinity-purified on nickel beads.

**Figure 5:**
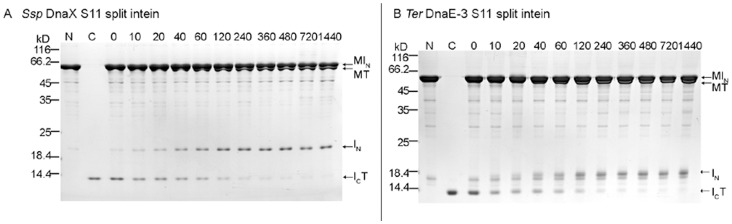
S11 split intein *trans*-splicing *in vitro*. For each split intein, purified C-protein (lane C) and N-protein (lane N), which are illustrated in Figure 4A, were mixed in a 1∶5 molar ratio and incubated under fixed set of conditions (25°C, 250 mM NaCl, 1 mM DTT, 20 mM Tris-HCl, pH 8.0). After specified number of minutes (specified on top), samples were taken and analyzed by SDS-PAGE and visualized by Coomassie blue staining. Positions are indicated for the precursor proteins (MIN and ICT), the splicing product (MT), and the excised N-intein (IN). The excised C-intein was too small (6 aa) to be seen in this analysis. Size markers (kD) are shown on the left.

**Figure 6:**
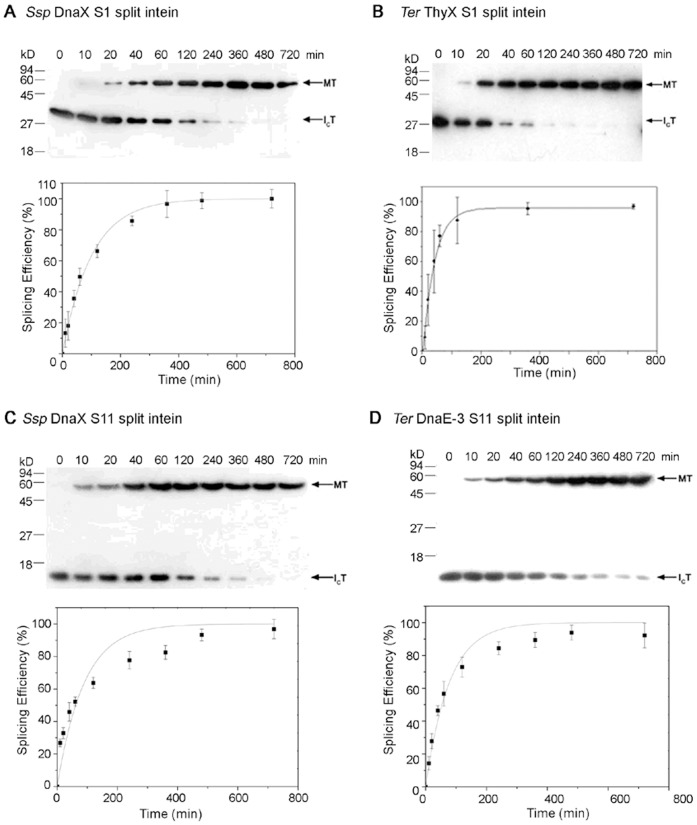
Kinetic analysis of *trans*-splicing *in vitro*. For each S1 or S11 split intein (specified in A to D), purified C-protein and N-protein (illustrated in Figures 3A and 4A) were mixed in a 1∶10 molar ratio and incubated under same conditions as in Figure 5. Samples were taken at different times (specified on top) and analyzed by Western blotting using an anti-T antibody. From the Western blot, relative amounts (band density) of the spliced protein (MT) and the C-protein (I_C_T) were estimated, and the splicing efficiency was calculated as MT/(MT+I_C_T). The splicing efficiency was plotted against the reaction time, which was used to estimate the reaction rate constant. All experiments were performed in triplicate, and error bars represent standard deviation.

We then mixed the two purified proteins and studied time course of the *trans*-splicing reaction *in vitro* (Figures 5 and 6). The C-protein and the N-protein were mixed at a molar ratio of 1∶5 (Figure 5) or 1∶10 (Figure 6), where the N-protein was added at a molar excess over the C-protein to achieve a pseudo-first order reaction regarding the C-protein, in order to estimate a rate constant of the *trans*-splicing reaction. In Figure 6, the reactions were performed at 20 and 200 micromolar concentrations for the C-protein and the N-protein, respectively. Amounts of the spliced protein (MT) and the remaining C-protein (I_C_T) were estimated on Western blots, the splicing efficiency was calculated as MT/(MT+I_C_T) and plotted against time, and the plot was fitted to the pseudo-first order reaction equation of *p* = P0(1−e^−kt^) to estimate the rate constant (*K*_obs_) [32]. The *Ssp* DnaX S1 split intein showed a rate constant of (1.7±0.1)×10^−4^ s^−1^ and a maximal splicing efficiency of 96%. The *Ter* ThyX S1 split intein showed a rate constant of (3.8±0.5)×10^−4 ^s^−1^ and a maximal splicing efficiency of 97%. The *Ssp* DnaX S11 split-intein showed a rate constant of (1.9±0.3)×10^−4 ^s^−1^ and a maximal splicing efficiency of 93%. The *Ter* DnaE-3 S11 split-intein showed a rate constant of (2.2±0.2)×10^−4 ^s^−1^ and a maximal splicing efficiency of 87%.

## Discussion

We successfully produced several new atypical split inteins that showed efficient *trans*-splicing activities when tested in *E. coli* and *in vitro*. This was achieved after modifying and testing over a dozen different natural inteins, which is summarized in Table 1. It is not easily predictable which natural intein can be converted into an atypical split intein and how. A few patterns can be seen in Table 1, which may be useful for future efforts to produce additional atypical split inteins. First, functional atypical split inteins were obtained only from mini-inteins that had a high level of *cis*-splicing activity, whereas none of the other seven mini-inteins that showed inefficient (<30%) or no *cis*-splicing activity gave rise to a functional S1 or S11 split intein. Second, mini-inteins capable of efficient *cis*-splicing usually produced a functional atypical split intein, with *Ter* RIR-1 intein being the only exception. Third, an efficient mini-intein could give rise to a functional S1 split intein, a functional S11 split intein, or both. Therefore, functional atypical split inteins may be more easily obtained by first producing mini-inteins capable of efficient *cis*-splicing. In this study, approximately 50% of the engineered mini-inteins showed efficient *cis*-splicing. The remaining mini-inteins failed to splice efficiently, probably due to an imprecise deletion of the putative endonuclease domain or an inappropriate insertion of the linker sequence containing hexahistidine. It may be possible to produce atypical split inteins directly from natural bi-functional inteins, but the resulting atypical split intein would contain the endonuclease domain that may cause undesirable complications in some applications.

**Table 1:** Summary of splicing activities of mini-inteins and split inteins in *E. coli*.

Natural inteins	Mini-inteins	S1 split inteins	S11 split inteins
*Ssp* DnaX	+++	+++	+++
*Ssp* GyrB	+++	+++	++
*Ter* DnaE-1	+	−	−
*Ter* DnaE-2	+	−	−
*Ter* DnaE-3	+++	−	+++
*Ter* DnaB-1	−	−	−
*Ter* RIR-1	++	−	−
*Ter* RIR-2	−	−	−
*Ter* RIR-3	−	−	−
*Ter* RIR-4	+	−	−
*Ter* ThyX	+++	+++	++
*Rma* DnaB	+++	+++	−
*CneA* PRP8	+++	−	−
*Tth* RIR	−	−	−

The level of splicing activity was estimated as the percentage of the precursor protein (or the C-protein for split inteins) that was converted to the spliced protein and presented as+++for >90%,++for 50–80%,+for 10–30%, and − for <5% (not detected).

It is interesting that only some of the efficiently *cis*-splicing mini-inteins gave rise to a functional S1 split intein and/or a functional S11 split intein, although all of these mini-intein sequences were split at identical or similar positions (see Figure 1). Crystal structures of different inteins are highly similar [5]–[7], even when the intein sequences are poorly conserved. The structural similarity is particularly high in intein’s splicing domain corresponding to a mini-intein, which includes intein’s catalytic center that contains the N- and C-terminal parts of the intein. For atypical split intein to catalyze a *trans*-splicing reaction, its two intein fragments (N-intein and C-intein) must associate and assemble properly to reconstitute a functional intein through intein fragment complementation. For S1 and S11 split inteins, it has been suggested that the larger intein fragment may form a nearly complete intein structure with a cavity or hole created by the absence of the smaller intein fragment [24], [25]. This cavity or hole is where the smaller intein fragment (which was expected to form β–strand) needs to insert or bind correctly, in order to form the functional catalytic center for *trans*-splicing. In those S1 and S11 split inteins that failed to *trans*-splice, the larger intein fragment might have not formed the appropriate cavity or hole in its structure. Alternatively, the smaller intein fragment might have failed to form the correct β–strand or to bind correctly in the hole/cavity on the larger intein fragment.

Under *in vitro* conditions used in this study, the four new atypical split inteins showed efficient *trans*-splicing. The *in vitro* conditions also allowed for analysis of reaction speed of the S1 and S11 split-inteins. Their apparent first-order rate constant ranged from ∼1.7×10^−4 ^s^−1^ to ∼3.8×10^−4 ^s^−1^, and these are comparable to or higher than those of previously reported atypical split inteins. Specifically, the previously reported *Ssp* GyrB S11 split intein showed a rate constant of ∼6.9×10^−5 ^s^−1^ [23], the previously reported *Ssp* DnaB S1 split intein showed a rate constant of ∼4.1×10^−5 ^s^−1^ [22], and an improved version of the *Ssp* DnaB S1 split intein showed a higher rate constant of ∼2.5×10^−3^ s^−1^ [33]. Differences of rate constants also exist among conventional split inteins where the intein fragments are more equal in size, which ranged from ∼10^−5^ s^−1^ for the naturally occurring *Ssp* DnaE split intein to ∼10^−2^ s^−1^ for the naturally occurring *Npu* DnaE split intein [34].

We have significantly expanded the availability of different atypical split inteins, and this may facilitate a more general use of protein-peptide *trans*-splicing, because different inteins have been known to behave differently when used in non-native host proteins. Although inteins are self-splicing elements, different inteins have co-evolved with different native host proteins and may therefore work differently when used in non-native host proteins. Inteins are also known to prefer their native extein amino acid residues immediately flanking the intein, presumably because these extein residues are at or near the splice sites and can potentially influence the intein’s catalytic center. For these reasons, different atypical split inteins derived from different natural inteins likely have different preferences of host proteins, although such preferences are difficult to determine. The increased availability of atypical split inteins may allow people to test several different inteins with a non-native host protein of interest, in order to find an intein that splices most efficiently with the protein of interest. The different atypical split inteins also have different native extein residues immediately flanking the intein. In particular, a nucleophilic residue at the +1 position immediately after the intein is required for the splicing function. Among the atypical split inteins of this study, some has cysteine at the +1 position, while others have serine at the +1 position. The availability of these atypical split inteins gives people more choices of where to insert intein in a host protein of interest. It also gives people more choices of what extra flanking residues to be included with intein for optimal splicing activity, so that one can minimize potential drawbacks caused by the extra flanking residues that will remain in the host protein after splicing. Furthermore, the new split inteins have an affinity tag sequence (hexahistidine) incorporated into the larger fragment of the intein, which serves as a removable tag for affinity purification of fusion proteins containing the intein fragment. Altogether, these engineered inteins are significant additions to the toolbox for many known applications of protein *trans*-splicing using atypical split inteins.
